# Perioperative management of liver surgery—review on pathophysiology of liver disease and liver failure

**DOI:** 10.1007/s10353-018-0522-4

**Published:** 2018-04-13

**Authors:** Lukas Gasteiger, Stephan Eschertzhuber, Werner Tiefenthaler

**Affiliations:** 1 Department of Anaesthesia and Intensive Care, Medical University of Innsbruck, Anichstraße 35, 6020 Innsbruck, Austria; 2 Department of Anaesthesia and Intensive Care, General Hospital Hall in Tirol, Hall in Tirol, Austria

**Keywords:** Chronic liver disease, Perioperative medicine, High risk patients, Coagulopathy

## Abstract

An increasing number of patients present for liver surgery. Given the complex pathophysiological changes in chronic liver disease (CLD), it is pivotal to understand the fundamentals of chronic and acute liver failure. This review will give an overview on related organ dysfunction as well as recommendations for perioperative management and treatment of liver failure-related symptoms.

## Introduction

The liver is the biggest intestinal organ and plays a central role in the homeostasis of different physiological systems including nutrition and drug metabolism, the synthesis of plasma proteins and haemostatic factors, as well as the elimination of different endogenous and exogenous substances [1]. Although the liver contributes with only 3% to total body weight, given its major role in homeostasis and high energy consumption, it receives 25% of total cardiac output (CO). Two vessels contribute to the perfusion of the liver. The majority (70%) of the hepatic perfusion is provided by the portal vein, which contributes 50% of the organ’s oxygen demand. The other 50% is provided by the hepatic artery, which makes up around 30% of total liver perfusion. Hepatic arterial blood flow is mainly dependent on the organ’s metabolic demands and controlled via autoregulatory mechanisms, whereas blood supply through the portal vein depends on the perfusion throughout the whole gastrointestinal tract and the spleen [2]. This unique, dual perfusion system provides constant perfusion rates and oxygen supply, which is crucial for adequate liver function. These high oxygen demands are reflected in a hepatic vein saturation of almost 30%.

The liver is also unique in its ability of regeneration, which allows the performance of major surgery including, amongst others, extended resections of liver tumours, living donations and so on. Many patients have normal liver function parameters when they present for liver surgery, especially when the reason for resection is metastasis or a benign liver tumour.

The most common causes of liver resections are the hepatocellular carcinoma (HCC) and the cholangiocellular carcinoma (CCC). Hepatocellular carcinoma (HCC) often develops in patients with underlying liver cirrhosis; many of these patients show signs of chronic liver dysfunction (CLD).

As explained previously, the liver plays a central role in a great deal of physiological systems. Therefore, in case of chronic liver dysfunction (CLD) or liver failure, several effects on other organ systems have to be expected. Consequently, the European Society of Anaesthesiology (ESA) as well as the European Society of Cardiology (ESC) classify liver resections and bile duct surgery as having a high risk for perioperative cardiac events, with an estimated 30-day cardiac event rate (cardiac death and myocardial infarction) of more than 5% [3].

## Pathophysiology of chronic liver disease:

### Circulation

In CLD, a hyperdynamic circulatory syndrome is observed, which is due to systemic vasodilatation that leads to considerable organ damage and complications (e.g. rupture of oesophageal and/or gastric varices, hepatic encephalopathy, ascites, and hepatopulmonary and hepato-renal syndrome) [4]. In the cirrhotic liver, a reduced bioavailibity of nitric oxide (NO) leads to portal hypertension by increasing the intrahepatic resistance, whereas in the splanchnic region the opposite, a massive vasodilatation, can be found [5].

The underlying causes for this progressive vasodilatory syndrome are attributed to an activation of vasoactive substances such as NO, cyclooxygenase derivatives, carbon monoxide and endogenous cannabinoids [6]. At the same time, both the vasoconstrictor and the sympathetic nervous system show a decreased activity.

The hyperdynamic syndrome is characterised by splanchnic vasodilatation, caused by elevated synthesis of NO due to enhanced activity of endothelial nitric oxide synthase (e-NOS) [7, 8]. Elevated CO and heart rate, decreased systemic vascular resistance (SVR) and low mean arterial blood pressure (MAP) are the consequences [9]. This endothelial dysfunction is of major importance regarding the prediction of adverse early hepatic events as well as mortality in CLD and liver cirrhosis [5].

The systemic vasodilatation leads to relative hypovolaemia, which is compensated by an increase in CO and, eventually, cirrhotic cardiomyopathy can be the consequence, culminating in deterioration of cardiac function and reduced CO. This leads to the disappearance of the hyperdynamic state. Cardiac hypertrophy, diastolic dysfunction and a prolonged QT-interval can also be found. In the absence of other cardiac diseases, echocardiographic findings of diastolic dysfunction and E/e’ ratio might be of diagnostic help [10].

### Renal function

Renal dysfunction is often seen in CLD, caused by the so-called hepatorenal syndrome (HRS). Still, the pathophysiology is not fully understood. Some data indicate that the reason might be the combination of increased CO and splanchnic vasodilatation leading to a decrease of renal perfusion and glomerular filtration rate (GFR) [11–13]. In response, raised levels of angiotensin II and antidiuretic hormone lead to an increase in renal vasoconstriction, thereby worsening renal perfusion. Due to reduced sodium excretion and increased water retention, a relative hyponatremia is found. Several meta-analyses indicated that the administration of terlipressin or noradrenaline might be a therapeutic option for HRS [14, 15].

### Lung function

Pulmonary function can be negatively influenced by pleural effusion, ascites and a high-standing diaphragm leading to atelectasis and impaired lung motion. This so-called hepatopulmonary syndrome (HPS) is often accompanied by portopulmonary hypertension (PPHTN).

The triad of intrapulmonary vasodilatation, increase in alveolar–arterial gradient and CLD is the typical manifestation of HPS. Profound hypoxemia may be the consequence [1, 16]. Perfusion lung scanning, contrast-enhanced echocardiography and pulmonary arteriography are possible diagnostic instruments to detect vascular abnormalities.

PPHTN, on the other hand, is characterised by an increase in intrapulmonary vasoconstriction and marked vascular remodelling that can cause right heart failure [16]. It is defined by portal hypertension (15 mm Hg) and mean pulmonary artery pressure >25 mm Hg. The severity of PPHTN is graded based on right heart catheterisation data. The prevalence is about 5%. PPHTN with a mean pulmonary pressure >40 mm Hg poses a very high intraoperative risk. It is to state that both the HPS and the PPHTN have severe prognostic implications [17–19].

### Coagulation

Coagulopathy is a major problem in CLD. It has a multifactorial aetiology (malnutrition, poor absorption of nutrients and impaired synthesis of coagulation factors) and varies depending on the underlying liver disease. Patients with HCC, per example, often have normal coagulation tests. The severity of the coagulation disorder serves as predictor for synthetic liver function, as most of the coagulation factors are produced in the liver. A low platelet count that may be caused by portal hypertension, splenic congestion or decreased hepatic release of thrombopoetin is an important prognostic factor. Moreover, it is regarded as an independent risk factor for considerable complications, postoperative liver insufficiency and mortality after major liver surgery [20]. Thrombocytopenia is often (at least partly) compensated by an increase of von Willebrand factor (VWF) levels.

As there is a lack of procoagulant and anticoagulant factors, prolonged standard coagulation tests (PT, PTT) poorly predict bleeding risk [21]. The CLD patient is in a so-called rebalanced haemostasis [21–24]. Recent studies indicate that thromboelastography (TEG) provides a better assessment of bleeding risk than conventional coagulation tests [25].

### Hepatic encephalopathy

In CLD, the decreased clearance of neurotoxins like ammonia, short-chain fatty acids and mercaptans causes these to accumulate and hepatic encephalopathy may develop. In the central nervous system, ammonia is metabolised to glutamine that can lead to cerebral oedema due to increased osmolality. A clear correlation between the grade of encephalopathy and ammonia concentration does not exist, but still, ammonia seems to play a central role in this disease pattern [26].

The severity of hepatic encephalopathy is graded from 0 (minimal hepatic encephalopathy) to 4 (coma) using the West Haven classification [27]. Gastrointestinal bleeding, sepsis, hyponatraemia and uraemia can aggravate the symptoms and predispose the patient to respiratory failure and aspiration [28]. The control of ammonia production and uptake in the gastrointestinal tract, as well as the increase of its elimination, are the corner stones in the management of hepatic encephalopathy. The non-absorbable disaccharide lactulose and enteral antibiotics (rifaximin and neomycin) have proven to be helpful in this context [29–31].

### Gastrointestinal system

Due to the combination of portal hypertension and the excessive retention of sodium and water, ascites can develop. This occurs in more than 50% of patients with cirrhosis within 10 years and is a predictor of poor prognosis [32, 33]. Sodium and water restriction are the first therapeutic options, followed by diuretic therapy and paracentesis [34]. Ascites removal can improve pulmonary function and reduce the risk of aspiration. Major complications of ascites are spontaneous bacterial peritonitis and HRS.

Nonetheless, the rapid drainage of ascites can be dangerous because it may lead to paracentesis-induced circulatory dysfunction (PICD), with increased risk of fast re-accumulation of the ascites, increased incidence of HRS and a higher mortality [35].

## Perioperative management of liver resection

It is obligatory that liver resection be performed under general anaesthesia. A variety of different issues have to be considered by the anaesthetist.

### Preoperative evaluation

The physical examination and preoperative testing should exclude an acute liver disease.

It should be kept in mind that increased values in so called liver function tests (LFT) like alanine aminotransferase (ALT) or aspartate aminotransferase (AST) mainly provide information regarding hepatocellular integrity rather than making statements about functionality [26]. As albumin and most coagulation factors are synthesised by the liver, they reflect liver function more precisely [36].

Laboratory testing should also include haemoglobin, white cell count, platelets count, electrolytes, glucose, blood urea nitrogen, creatinine, prothrombin time (PT)/international normalized ratio (INR), partial thromboplastin time (PTT), fibrinogen, AST, ALT, albumin, cholinesterase and bilirubin. As LFT are profoundly increased in any acute liver failure (acute viral, hepatitis, ischemic hepatitis, acute drug- or toxin-induced liver injury), they should be examined regularly in any patient with a known liver disease. In such cases, or in case of exacerbations of a known CLD combined with any other liver disease-associated organ failure, elective liver surgery should be postponed [37].

There are a few scoring systems that help to decide whether liver surgery can be performed safely in patients with CLD. For example, an ASA (American Society of Anesthesiologists) score of 4 serves as an independent risk factor for mortality [26, 38]. The Child–Turcotte–Pugh classification (CTP) still strongly correlates with mortality in surgery of patients with CLD and should be incorporated in the decision. Patients with a CTP score less than 8 should undergo surgery with acceptable risk [39]. The Model of End-Stage Liver Disease (MELD) score was developed in order to predict short-term survival for patients who are on the waiting list for liver surgery, even though in past years it was increasingly used to predict perioperative risk in patients with CLD or end-stage liver disease (ESLD). There is no clear cut-off level for elective surgery. A score between 10 and 15 is associated with increased perioperative risk. With a value greater than 15, elective surgery should be postponed [40, 41].

In a healthy liver (normal parenchyma) with normal liver function, a rest volume of 25–30% after liver resection normally allows adequate postoperative liver function. Laboratory testing of INR, bilirubin and albumin give information about the liver function, but they cannot predict the functional reserve of the liver. Neither are the indocyanin green (ICG) clearance or monoethylglycinexylidide synthesis (MEGX-test) tests able to accurately predict liver function after resection [9].

### Other organ systems

As described previously, CLD can have severe impact on many organ systems. The following abstract gives a short summary of important side effects and disorders that need to be checked preoperatively in any CLD patient.

#### Encephalopathy

Hepatic encephalopathy must be excluded by physical examination.

#### Haematological and coagulation disorders

PT/INR, PTT, haemoglobin and platelet count need to be controlled on a regular basis in case of CLD. The latter should always be > 50,000/μL. Administration of vitamin K should be mentioned. The administration of fibrinogen to maintain a fibrinogen level >200 mg/dl should be mandatory. There is no evidence for administration of fresh frozen plasma to correct INR. TEG analysis can be of great help [42].

#### Cardiovascular system

In patients scheduled for liver surgery, an ECG should always be performed. Given the massive impact CLD and cirrhosis can have on the circulation, cardiac function should be assessed in particular. Left ventricular as well as right ventricular dysfunction must be expected due to hyperdynamic circulation, cirrhotic cardiomyopathy and PPHTN (see pathophysiology of chronic liver disease Circulation). Severe PPHTN is an indication for liver transplantation and obviously a contraindication for elective liver resection. Apart from these liver-related problems, other causes of cardiomyopathy (CMP) can occur, such as alcoholic CMP or CMP related to hemosiderosis [43].

#### Pulmonary system

As underlined in the introduction, patients may suffer from dyspnoea due to a high-standing diaphragm caused by ascites or by pleural effusions. Intrapulmonary vasodilatation caused by HPS can cause a massive ventilation–perfusion mismatch with consequent hypoxaemia. Patients with HPS must be differentiated from those with pulmonary hypertension generated from high CO, volume overload or venous hypertension. For the management of patients with HPS, prostacyclin analogues, phosphodiesterase inhibitors or endothelin antagonists are used in our institution. Patients with severe HPS are evaluated for liver transplantation and elective surgery is not indicated.

#### Portal hypertension

The existence and the amount of ascites must be evaluated. Ascites should be managed as described above and paracentesis prior to surgery should be performed if other organs (e.g. lungs) are affected negatively.

#### Renal system

CLD patients are at risk of developing HRS with retention of sodium and free water. There is also a certain risk for other causes of renal dysfunction such as parenchymal damage, nephrotoxicity, sepsis and hypovolaemia. Urine output, creatinine and urea levels should be monitored closely and in the perioperative period. Hyperkalaemia, acidosis and exposure to nephrotoxins (e.g. aminoglycosides, NSAID, contrast agents) should be avoided.

In case of HRS type I, the administration of albumin should be taken into account. The administration of vasoconstrictor agents like norepinephrine or terlipressin has been proven in case of HRS type I [44].

#### Electrolyte abnormalities

Hyponatraemia develops slowly in patients with cirrhosis. Unless the sodium levels drop below 120 mEq/l or if neurologic symptoms develop, it should not be corrected. If correction is necessary, it should be done slowly to avoid central pontine myelinolysis. Hypokalaemia and metabolic alkalosis can develop and may worsen hepatic encephalopathy. Hypokalaemia should be corrected preoperatively.

## Intraoperative management

### Haemodynamic management

In addition to standard anaesthesia monitoring (ECG, pulse oximetry, temperature monitoring, relaxometry, bladder catheter), haemodynamic monitoring should include the insertion of an arterial catheter to continuously measure arterial blood pressure, and a central venous catheter (CVC) to measure central venous pressure (CVP). The insertion of transesophageal echocardiography (TEE) may be of concern in patients with oesophageal varices, even though it may be beneficial for haemodynamic and volume monitoring.

The insertion of a pulmonary arterial catheter (PAC) can be considered in patients with decreased cardiac function. Alternatively, the application of a continuous haemodynamic monitoring system (e.g. PICCO™, Pulsion Medical Systems, Munich, Germany) could be indicated.

Given the potential for massive haemorrhage during hepatic preparation and resection, the insertion of large-bore venous catheters is mandatory. In our institution, we normally place a four-lumen CVC and second large-bore CVC with a port for a pulmonary arterial catheter. In patients with decreased renal function, an additional Quinton catheter for eventual postoperative hemofiltration may be useful.

### Volume management

The amount of blood transfusions correlates directly with postoperative complications and mortality [45]. Therefore, both surgical as well as anaesthesiologic management should aim do minimise blood loss.

The two major reasons for bleeding in liver resections are surgical lesions in extrahepatic liver veins and bleeding due to parenchyma transection. Blood pressure in hepatic sinusoids depends on hepatic venous pressure, which also correlates with CVP.

Lowering CVP is a simple but effective method to reduce blood loss during liver resections [46, 47]. Although the value and necessity of low CVP is still discussed, we aim for a low CVP during preparation and resection to reduce blood loss and facilitate surgical handling.

Minimizing intravenous volume infusion is also an effective tool to decrease CVP. If this is not sufficient, intravenous use of nitro glycerine can be used in order to reduce CVP. Volume and blood loss must be monitored and corrected continuously. Vasopressor therapy is often necessary to maintain adequate blood pressure levels. Lowering PEEP during resection to an acceptable minimum (i.e. 5 cmH2O) can help to reduce CVP. According to the literature, low CVP-assisted liver resections do not lead to an increase in acute kidney injury (AKI) [48].

Volume status needs to be evaluated repeatedly, because hypovolaemia can mediate organ dysfunction trough impaired systemic tissue perfusion. But one has to be careful, because hypervolemia increases oedema formation [49].

Urine output must be monitored continuously as an intraoperative oliguria <0.3 ml/kg/h is associated with an increased risk of postoperative AKI [50].

For fluid replacement we use colloids (Gelofusin™, B. Braun Melsungen AG, Melsungen, Germany) and balanced crystalloid fluids in our hospital.

Depending on blood loss and coagulation tests (TEG), we use fresh frozen plasma and coagulation factors for coagulation therapy. The use of antifibrinolytic agents (tranexamic acid) is indicated in operations with major blood loss or signs of hyperfibrinolysis in the TEG.

Given the negative impact of hypothermia on coagulation, it is crucial to monitor and control the patient’s temperature. Normothermia should be maintained by using extern-warming systems (e.g. Bair Hugger™, 3M, St. Paul, United States).

## Conclusion

This review demonstrates that patients undergoing liver surgery pose a significant challenge to treating physicians in the perioperative period. Due to the improvement of surgical techniques, the “liver patient” is becoming more and more complex, confronting anaesthetists and intensive care personnel with difficult intra- and postoperative courses, and considerable multiorgan disorders. The cornerstones of an optimal management are careful selection of the patients, appropriate monitoring and protection of the liver and other vital organs.
